# Correction: Effective methods for the inactivation of *Francisella tularensis*

**DOI:** 10.1371/journal.pone.0226125

**Published:** 2019-12-02

**Authors:** The PLOS ONE Staff

Fig 5 is incorrect. Please see the correct Fig 5 here. The publisher apologizes for the error.

**Fig 5:**
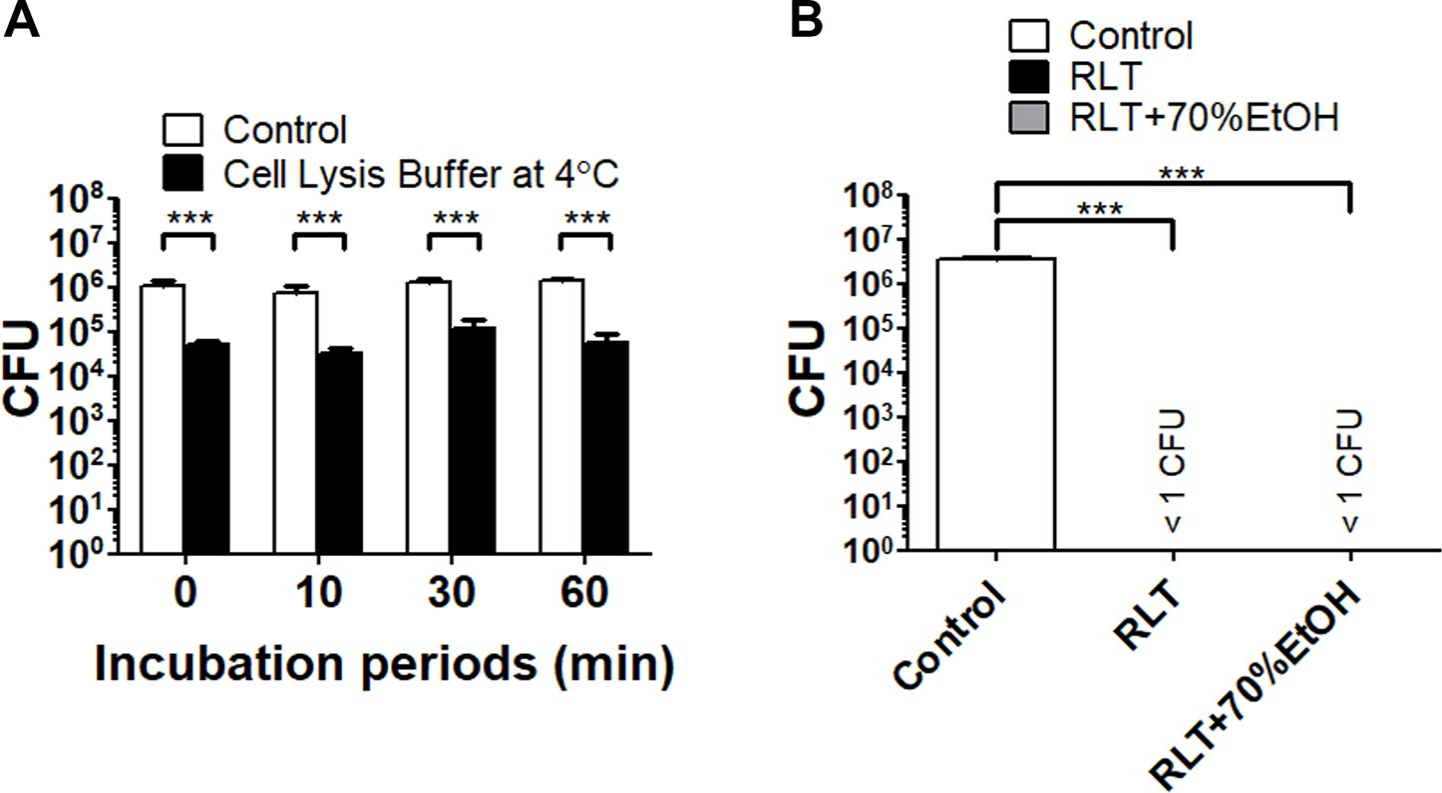
The viability of *F*. *tularensis* SCHU P9 after treatments of commercial products. Bacterial viability was evaluated after the treatment using Cell Lysis Buffer (Cell Signaling Technology) and the RLT buffer supplied by RNeasy mini kit (Qiagen Ltd.,). (A) Bacteria suspended in Cell Lysis Buffer and CDM (control) were incubated at 4°C for the indicated time. (B) Bacterial pellets after the centrifugation at 12,000 × g for 2 min at 4°C were suspended in RLT buffer alone, the mixture of RLT buffer and 70% ethanol, and CDM (control). The samples were incubated at room temperature for 10 min. All incubated samples were centrifuged at 12,000 × g for 2 min at 4°C and the pellets were suspended in CDM. the mean CFU ± SD of control and the treatment samples are shown. Statistical significance was determined by two-way ANOVA (A) and one-way ANOVA (B) with the *post hoc* test (***p < 0.001).
