# Supplementary figures and images for: Effective methods for the inactivation of Francisella tularensis
Source: PLoS One. 2019 Nov 14;14(11):e0225177. doi: 10.1371/journal.pone.0225177 (PMC6855423; doi:10.1371/journal.pone.0225177)

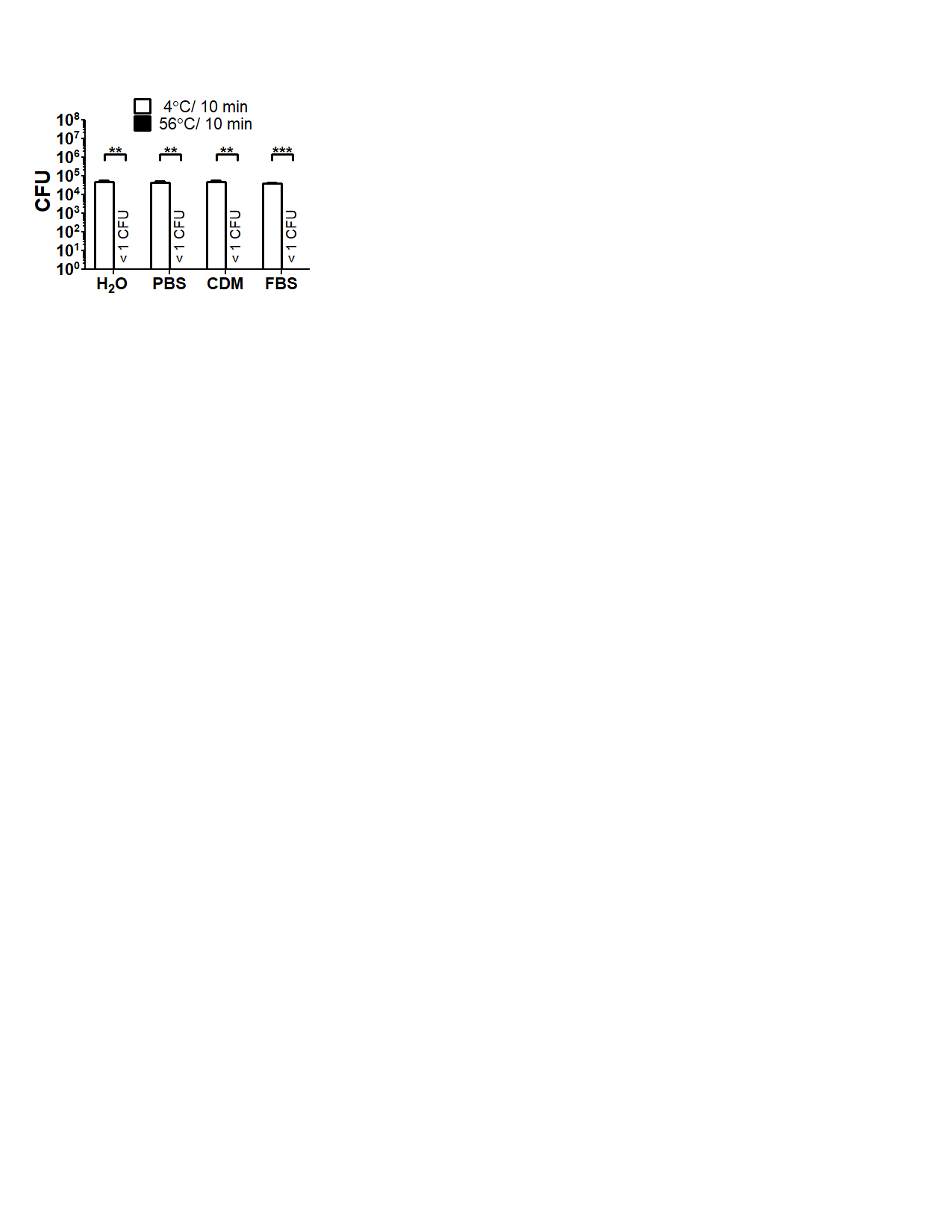

Supplement: S1 Fig — Bacterial suspensions were prepared with deionized water, CDM, PBS, and undiluted FBS. The samples were heated at 56°C for 10 min and then immediately cooled on ice. The black and white bars indicate the CFU numbers of the treated and control samples, respectively. Statistical significance was determined by two-way ANOVA with a post hoc test (**p < 0.01 and ***p < 0.001). (TIF) [file pone.0225177.s001.tif]
